# *Rickettsia typhi* and *R*. *felis* in Rat Fleas (*Xenopsylla cheopis*), Oahu, Hawaii 

**DOI:** 10.3201/eid1410.080571

**Published:** 2008-10

**Authors:** Marina E. Eremeeva, Wesley R. Warashina, Michele M. Sturgeon, Arlene E. Buchholz, Gregory K. Olmsted, Sarah Y. Park, Paul V. Effler, Sandor E. Karpathy

**Affiliations:** Centers for Disease Control and Prevention, Atlanta, Georgia, USA (M.E. Eremeeva, M.M. Sturgeon, S.E. Karpathy); Hawaii Department of Health, Honolulu, Hawaii, USA (W.R. Warashina, A.E. Buchholz, G.K. Olmsted, S.Y. Park, P.V. Effler)

**Keywords:** Murine typhus, Rickettsia felis, Rickettsia typhi, molecular assays, Hawaii, dispatch

## Abstract

*Rickettsia typhi* (prevalence 1.9%) and *R*. *felis* (prevalence 24.8%) DNA were detected in rat fleas (*Xenopsylla cheopis*) collected from mice on Oahu Island, Hawaii. The low prevalence of *R. typhi* on Oahu suggests that *R. felis* may be a more common cause of rickettsiosis than *R. typhi* in Hawaii.

Murine typhus is a febrile zoonotic disease caused by *Rickettsia typhi*. The classic view is that *R*. *typhi* circulates among rats (*Rattus rattus* or *R*. *norvegicus*) and rat fleas (*Xenopsylla cheopis*) (*1*,*2*), although other rodents and their ectoparasites have been implicated in maintenance of *R*. *typhi* in nature. Humans become infected when they visit disease-endemic areas infested with rats and acquire infection by inhalation or by self-inoculating infected fleas or flea feces into skin.

The most recent outbreak of murine typhus in Hawaii occurred in 2002 with 47 cases reported on 5 islands (*3*). Concomitantly, an increase occurred in the mouse population on the island of Maui, which reported 35 human cases. Peak occurrence of murine typhus in Hawaii was in 1944 with 186 cases reported, of which 80% occurred on the island of Oahu (*4*). Previous serologic surveys in Hawaii have identified antibodies reactive with *R*. *typhi* antigen in the Polynesian rat (*R*. *exulans*), black rat (*R*. *rattus*), Norway rat (*R*. *norvegicus*), and house mouse (*Mus musculus*) (*3*,*4*). The Indian mongoose (*Herpestes auropunctatus*) was also identified as a potential reservoir; however, its role has not yet been evaluated. We conducted a molecular survey of fleas in Oahu to characterize the prevalence and identity of rickettsiae in Hawaii.

## The Study

*M*. *musculus* mice were collected during rodent population studies in the leeward and southeast parts of Oahu during the summers of 2004, 2006, and 2007 (Figure). Fleas were combed from each animal, identified as *X*. *cheopis* by using standard taxonomic keys, and kept frozen at –70°C until they were sent to the Centers for Disease Control and Prevention (Atlanta, GA, USA) for further analyses. Mice were humanely killed; only brains were removed and frozen.

**Figure:**
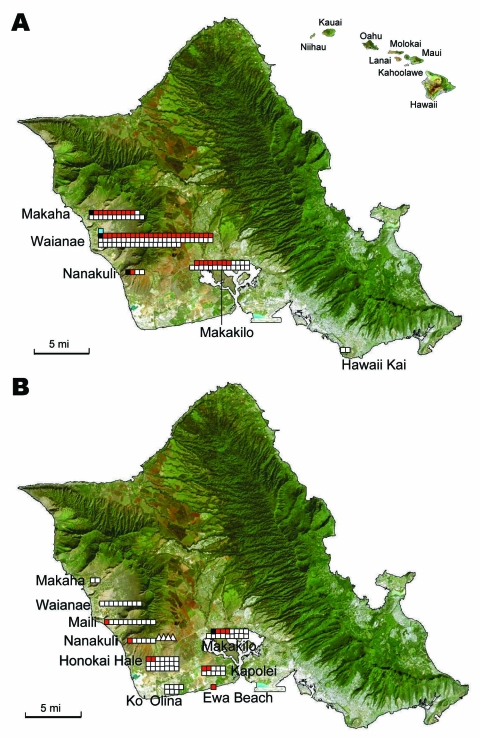
Detection of *Rickettsia typhi* and *R. felis* DNA in *Xenopsylla cheopis* trapped in Oahu, Hawaii, in A) 2004 and B) 2006 and 2007. Hawaii is shown in the inset. Symbols correspond to sites of sample collection. White squares, collections in 2004 and 2006 of fleas negative for *R*. *felis* and *R*. *typhi*; white triangles, collections in 2007 of fleas negative for *R*. *felis* and *R*. *typhi*; red squares, fleas positive for *R*. *felis*; black squares, fleas positive for *R*. *typhi*; blue squares, fleas positive for both *R*. *typhi* and *R*. *felis.* Maps were obtained from www.hear.org/starr/maps/stock/landsat.htm

DNA was isolated from each flea by using the Biomek 2000 Laboratory Automation workstation (Beckman, Fullerton, CA, USA) and reagents from the Wizard Prep kit (Promega, Madison, WI, USA) (*5*). DNA from 20 mg of mouse brain tissue was isolated by using the QiaAmp Mini kit (QIAGEN, Valencia, CA, USA).

Detection of *R*. *felis* and *R*. *typhi* DNA was conducted by using a TaqMan assay for the citrate synthase (*glt*A) gene of *Rickettsia* spp. (forward primer: 5′-GATTTTTTAGAAGTGGCATATTTG-3′; reverse primer: 5′-GGKATYTTAGCWATCATTCTAATAGC-3′) and species-specific probes (*R*. *typhi*: 5′-CalRed610-TT(T)A(C)TA(C)A(A)AG(A)T(T)G(C)T(C)A-BHQ2-3′; *R*. *felis*: 5′-Cy5-CTA(C)GGA(G)AATT(G)CCA-BHQ3-3′); locked nucleic acid bases, shown in parentheses, were incorporated to improve probe binding. Specificity of probes was tested by using DNA of *R*. *prowazekii*, *R*. *typhi*, and 23 spotted fever group rickettsial isolates. The Brilliant Q PCR core reagent kit (Stratagene, La Jolla, CA, USA) and an *i*Cycler (Bio-Rad, Hercules, CA, USA) were also used. Positive control plasmids contained a 265-bp target fragment from *R*. *typhi* strain Wilmington and *R*. *felis* strain LSU.

DNA from 210 *X*. *cheopis* fleas was examined, including 122 fleas collected from 61 mice trapped in 2004, 84 fleas from 55 mice trapped in 2006, and 4 fleas from 2 mice trapped in 2007 (Table). Victor Tin Cat Repeating Mouse Traps (Woodstream Corp., Lititz, PA, USA) were located in 10 communities on the leeward and southeast parts of Oahu, the former representing areas where typhus cases are most frequently diagnosed on this island. The largest collections were obtained from Waianae (36.6% of flea specimens), Makakilo (20%), and Makaha (11.9%); only 1 sample each was available from Ewa Beach and Hawaii Kai. An average of 1.8 (median 1) fleas was collected from each animal.

**Table:** Prevalence of *Rickettsia felis* and *R*. *typhi* in *Xenopsylla cheopis* fleas by PCR, Oahu, Hawaii*

Location	No. (%) positive collected in 2004						No. (%) positive collected in 2006				
	Mice	Fleas	*R*. *felis*	*R*. *typhi*	*R*. *felis* and *R*. *typhi*		Mice	Fleas	*R*. *felis*	*R*. *typhi*	*R* *felis* and *R*. *typhi*
Ewa Beach	0	0	NA	NA	NA		1	1	1	0	0
Hawaii Kai	1	2	0	0	0		0	0	NA	NA	NA
Honokai Hale	0	0	NA	NA	NA		12	21	2 (10)	0	0
Kapolei	0	0	NA	NA	NA		7	10	2 (20)	0	0
Ko Olina	0	0	NA	NA	NA		3	7	0	0	0
Maili	0	0	NA	NA	NA		7	11	1 (9)	0	0
Makaha	12	23	9 (39)	1 (4.3)	0		2	2	0	0	0
Makakilo	17	25	8 (32)	0	0		13	17	3 (18)	1 (6)	0
Nanakuli	3	4	1 (25)	1 (25)	0		6	6	1(17)	0	0
Waianae	28	68	24 (35)	1(1.5)	1 (1.5)		4	9	0	0	0
Subtotal	61	122	42 (34)	3 (3)	1 (0.8)		55	84	10(12)	1 (1.2)	0
Total†	118	210							52 (25)	4 (1.9)	1 (0.05)

*NA, not available (no samples were collected). †Includes 2 mice and 4 fleas collected in 2007 from Nanakuli; these samples were PCR negative.

Four fleas (1.9%, n = 210) contained only *R*. *typhi* DNA, and 52 (24.8%) fleas contained only *R*. *felis* DNA. The amplicon sequences were identical to homologous sites of *R*. *typhi glt*A (AE017197) or *R*. *felis glt*A (CP000053). One flea contained *R*. *felis* and *R*. *typhi* DNA. This result was confirmed by cloning of 4 replicate amplicons and sequencing of 24 randomly selected clones. Both DNAs were confirmed to be present in each amplicon. The highest rates of fleas infected with rickettsial agents were detected in Makaha (44%, n = 23), Waianae (38%, n = 68), and Makakilo (32%, n = 25) during the 2004 collection. *R*. *typhi* was detected in 4 sites (Makaha, Makakilo, Nanakuli, and Waianae). All DNAs extracted from brain tissues of 55 mice collected in 2006 were PCR negative for *R*. *felis* and *R. typhi*.

## Conclusions

We report molecular detection and identification of *R*. *typhi* associated with rat fleas (*X*. *cheopis*) collected from house mice (*M*. *musculus*) in western Oahu, Hawaii. *R*. *felis*, the etiologic agent of cat flea rickettsiosis, was also found associated with rat fleas collected from house mice.

The role of commensal rats and their fleas is often regarded as axiomatic for maintenance of murine typhus (*1*,*2*). However, other rodents and their ectoparasites have been implicated as alternative competent reservoirs and vectors of *R*. *typhi,* respectively (*1*,*5*). House mice are highly susceptible to experimental infection with *R*. *typhi*, which may establish a persistent intracerebral infection lasting for up to 5 months and is excreted in the urine (*6*). A previous study reported house mice naturally infected with *R*. *typhi* in the state of Georgia (*7*); however, no PCR-positive mice were detected in our study. Eruptions of mouse populations in the absence of rats have been implicated in several outbreaks of murine typhus (*1*); however, these observations were not supported by laboratory data. Early reports relied mostly on isolation of rickettsiae from tissue or fleas and serosurveys that did not necessarily provide accurate speciation of rickettsial isolates in the absence of precise molecular characteristics. Recent surveillance reports applying PCR and sequencing for detection and identification of rickettsiae have also detected *R*. *typhi* DNA in *X*. *cheopis*, *Leptosylla segnis*, and *Ctenocephalides felis* fleas in different parts of the world (*2*,*5*,*8*).

*R*. *felis* has been detected in many countries, primarily associated with *C*. *felis* fleas parasitizing cats, dogs, or opossums (*2*), although *R*. *felis* is rarely detected in cats or opossums. Surprisingly, *R*. *felis* may be present in rat fleas (*X*. *cheopis*) as demonstrated here and in a recent report from Indonesia (*9*). The prevalence of *R*. *felis* ranges from 5% to 45.8% for large collections of fleas, sometimes up to 100% when small collections are tested (*8*,*10*,*11*), and is often higher when compared with the prevalence of *R*. *typhi*, as in our study. *R*. *felis* has also been detected in *Anomiopsyllus nudata* collected on white-throated woodrats (*Neotoma albigula*) (*12*). Co-infection with *R*. *felis* and *R*. *typhi* in fleas has been reported only in experimentally infected fleas (*13*). However, it is not known if either pathogen has any advantage for acquisition, life-long persistence, or transmission by fleas.

Murine typhus caused by *R*. *typhi* has been considered to be the only rickettsiosis present in Hawaii, but our data indicate that a second flea-borne rickettsia, *R*. *felis*, circulates in areas on Oahu where murine typhus is endemic. Clinical symptoms for cat flea rickettsiosis (CFR) are not agent specific and, as for other rickettsioses, include fever, headache, and rash. Antibodies against *R*. *felis* variably cross-react with *R*. *typhi*, *R*. *rickettsii*, and other spotted fever group rickettsia antigens (*14*). Consequently, *R*. *felis* infection in humans can be misdiagnosed or missed without *R*. *felis* antigen testing. Only a handful of cases of CFR have been reported worldwide, and only 8 cases have been specifically confirmed by PCR (*14*,*15*). The Hawaii State Department of Health reported a mean of 4.2 cases annually from 1992 through 2001 and a mean of 20 cases annually through 2006, but an outbreak of 47 cases occurred in 2002 (*3*). Because all cases of murine typhus in Hawaii were diagnosed by using potentially cross-group reactive serologic tests and not specific molecular or serologic tests, it is difficult to exclude or confirm if humans have CFR or what the relative prevalence of the 2 rickettsial diseases may be in Hawaii.

Since 25% of the fleas removed from mice were positive for only *R*. *felis* DNA, this pathogen may pose a serious risk to human health in Oahu. Further studies are warranted to establish the true human prevalence of murine typhus and cat flea rickettsiosis in Hawaii, to define the clinical spectrum of these infections with more specific confirmatory diagnostic tests, and to establish the role of fleas and different rodents in the epidemiology of the 2 diseases.

## References

[1] Traub R, Wisseman CL Jr, Farhang-Azad A. The ecology of murine typhus—a critical review. Trop Dis Bull. 1978;75:237–317. 705902

[2] Azad AF, Radulovic S, Higgins JA, Noden BH, Troyer JM. Flea-borne rickettsioses: ecologic considerations. Emerg Infect Dis. 1997;3:319–27. 9284376 10.3201/eid0303.970308 PMC2627639

[3] Centers for Disease Control and Prevention. Murine typhus—Hawaii, 2002. MMWR Morb Mortal Wkly Rep. 2003;52:1224–6. 14681594

[4] Manea SJ, Sasaki DM, Ikeda JK, Bruno PP. Clinical and epidemiological observations regarding the 1998 Kauai murine typhus outbreak. Hawaii Med J. 2001;60:7–11. 11272443

[5] Loftis AD, Reeves WK, Szumlas DE, Abbassy MM, Helmy IM, Moriarty JR, Surveillance of Egyptian fleas for agents of public health significance: *Anaplasma, Bartonella, Coxiella, Ehrlichia, Rickettsia*, and *Yersinia pestis.* Am J Trop Med Hyg. 2006;75:41–8. 16837707

[6] Philip CB, Parker RR. The persistence of the viruses of endemic (murine) typhus, Rocky Mountain spotted fever, and boutonneuse fever in tissues of experimental animals. Public Health Rep. 1938;53:1246–51.

[7] Brigham GD, Pickens EG. A strain of endemic typhus fever virus isolated from a house mouse (*Mus musculus musculus*). Public Health Rep. 1943;3:135–6.

[8] de Sousa R, Fournier PE, Santos-Silva M, Amaro F, Bacellar F, Raoult D. Molecular detection of *Rickettsia felis, Rickettsia typhi* and two genotypes closely related to *Bartonella elizabethae.* Am J Trop Med Hyg. 2006;75:727–31. 17038702

[9] Jiang J, Soeatmadji DW, Henry KM, Ratiwayanto S, Bangs MJ, Richards AL. *Rickettsia felis* in *Xenopsylla cheopis*, Java, Indonesia. Emerg Infect Dis. 2006;12:1281–3. 16965716 10.3201/eid1208.060327 PMC3291232

[10] Horta MC, Labruna MB, Pinter A, Linardi PM, Schumaker TT. Rickettsia infection in five areas of the state of São Paulo, Brazil. Mem Inst Oswaldo Cruz. 2007;102:793–801. 10.1590/S0074-02762007000700003 18094887

[11] Bitam I, Parola P, de la Cruz KD, Matsumoto K, Baziz B, Rolain JM, First molecular detection of *Rickettsia felis* in fleas from Algeria. Am J Trop Med Hyg. 2006;74:532–5. 16606979

[12] Stevenson HL, Labruna MB, Montenieri JA, Kosoy MY, Gage KL, Walker DH. Detection of *Rickettsia felis* in a New World flea species, *Anomiopsyllus nudata* (Siphonaptera: Ctenophthalmidae). J Med Entomol. 2005;42:163–7. 10.1603/0022-2585(2005)042[0163:DORFIA]2.0.CO;2 15799525

[13] Noden BH, Radulovic S, Higgins JA, Azad AF. Molecular identification of *Rickettsia typhi* and *R. felis* in co-infected *Ctenocephalides felis* (Siphonaptera: Pulicidae). J Med Entomol. 1998;35:410–4. 9701920 10.1093/jmedent/35.4.410

[14] Raoult D, La Scola B, Enea M, Fournier P-E, Roux V, Fenollar F, A flea-associated *Rickettsia* pathogenic for humans. Emerg Infect Dis. 2001;7:73–81. 11266297 10.3201/eid0701.010112 PMC2631683

[15] Oteo JA, Portillo A, Santibáñez S, Blanco JR, Pérez-Martínez L, Ibarra V. Cluster of cases of human *Rickettsia felis* infection from southern Europe (Spain) diagnosed by PCR. J Clin Microbiol. 2006;44:2669–71. 10.1128/JCM.00366-06 16825412 PMC1489505

